# Supplementary material for: COZOID: contact zone identifier for visual analysis of protein-protein interactions
Source: BMC Bioinformatics. 2018 Apr 6;19:125. doi: 10.1186/s12859-018-2113-6 (PMC5889581; doi:10.1186/s12859-018-2113-6)
Supplement: Supplementary file 2 — Software build. Executable binary file of the software tool. (ZIP 118,784 kb) [file 12859_2018_2113_MOESM2_ESM.zip › cozoid/java/docs/beansbinding-1.2.1-doc.zip!/org/jdesktop/beansbinding/BindingGroup.html]

BindingGroup (Beans Binding)


---


|  |  |  |
| --- | --- | --- |
| **Overview** \| **Package** \| **Class** \| **Use** \| **Tree** \| **Deprecated** \| **Index** \| **Help** | |  |
| **PREV CLASS**   **NEXT CLASS** | **FRAMES**    **NO FRAMES**     **All Classes** |  |
| SUMMARY: NESTED \| FIELD \| CONSTR \| METHOD | DETAIL: FIELD \| CONSTR \| METHOD |  |


---


## org.jdesktop.beansbinding Class BindingGroup

```
java.lang.Object
  org.jdesktop.beansbinding.BindingGroup
```

---

``` public class BindingGroup extends java.lang.Object ```

`BindingGroup` allows you to create a group of `Bindings`
and operate on and/or track state changes to the `Bindings` as
a group.

---

**Constructor Summary**

`BindingGroup()`
  
          Creates an empty `BindingGroup`.


| **Method Summary** | |
| --- | --- |
| `void` | `addBinding(Binding binding)`             Adds a `Binding` to this group. |
| `void` | `addBindingListener(BindingListener listener)`             Adds a `BindingListener` to be notified of all `BindingListener` notifications fired by any `Binding` in the group. |
| `void` | `bind()`             Calls `bind` on all unbound bindings in the group. |
| `Binding` | `getBinding(java.lang.String name)`             Returns the `Binding` in this group with the given name, or `null` if this group doesn't contain a `Binding` with the given name. |
| `BindingListener[]` | `getBindingListeners()`             Returns the list of `BindingListeners` registered on this group. |
| `java.util.List<Binding>` | `getBindings()`             Returns a list of all `Bindings` in this group. |
| `void` | `removeBinding(Binding binding)`             Removes a `Binding` from this group. |
| `void` | `removeBindingListener(BindingListener listener)`             Removes a `BindingListener` from the group. |
| `void` | `unbind()`             Calls `unbind` on all bound bindings in the group. |

**Methods inherited from class java.lang.Object**

`clone, equals, finalize, getClass, hashCode, notify, notifyAll, toString, wait, wait, wait`

**Constructor Detail**

### BindingGroup

```
public BindingGroup()
```

:   Creates an empty `BindingGroup`.


**Method Detail**

### addBinding

```
public final void addBinding(Binding binding)
```

:   Adds a `Binding` to this group.

    :   **Parameters:**: `binding` - the `Binding` to add **Throws:**: `java.lang.IllegalArgumentException` - if the binding is null, is a managed binding, if the group already contains this binding, or if the group already contains a binding with the same (`non-null`) name

---


### removeBinding

```
public final void removeBinding(Binding binding)
```

:   Removes a `Binding` from this group.

    :   **Parameters:**: `binding` - the `Binding` to remove **Throws:**: `java.lang.IllegalArgumentException` - if the binding is null or if the group doesn't contain this binding

---


### getBinding

```
public final Binding getBinding(java.lang.String name)
```

:   Returns the `Binding` in this group with the given name,
    or `null` if this group doesn't contain a `Binding`
    with the given name.

    :   **Parameters:**: `name` - the name of the `Binding` to fetch **Returns:**: the `Binding` in this group with the given name, or `null` **Throws:**: `java.lang.IllegalArgumentException` - if `name` is `null`

---


### getBindings

```
public final java.util.List<Binding> getBindings()
```

:   Returns a list of all `Bindings` in this group. Order is undefined.
    Returns an empty list if the group contains no `Bindings`.

    :   **Returns:**: a list of all `Bindings` in this group.

---


### bind

```
public void bind()
```

:   Calls `bind` on all unbound bindings in the group.

---


### unbind

```
public void unbind()
```

:   Calls `unbind` on all bound bindings in the group.

---


### addBindingListener

```
public final void addBindingListener(BindingListener listener)
```

:   Adds a `BindingListener` to be notified of all `BindingListener`
    notifications fired by any `Binding` in the group. Does nothing if
    the listener is `null`. If a listener is added more than once,
    notifications are sent to that listener once for every time that it has
    been added. The ordering of listener notification is unspecified.

    :   **Parameters:**: `listener` - the listener to add

---


### removeBindingListener

```
public final void removeBindingListener(BindingListener listener)
```

:   Removes a `BindingListener` from the group. Does
    nothing if the listener is `null` or is not one of those registered.
    If the listener being removed was registered more than once, only one
    occurrence of the listener is removed from the list of listeners.
    The ordering of listener notification is unspecified.

    :   **Parameters:**: `listener` - the listener to remove **See Also:**: `addBindingListener(org.jdesktop.beansbinding.BindingListener)`

---


### getBindingListeners

```
public final BindingListener[] getBindingListeners()
```

:   Returns the list of `BindingListeners` registered on this
    group. Order is undefined. Returns an empty array if there are
    no listeners.

    :   **Returns:**: the list of `BindingListeners` registered on this group **See Also:**: `addBindingListener(org.jdesktop.beansbinding.BindingListener)`


---


|  |  |  |
| --- | --- | --- |
| **Overview** \| **Package** \| **Class** \| **Use** \| **Tree** \| **Deprecated** \| **Index** \| **Help** | |  |
| **PREV CLASS**   **NEXT CLASS** | **FRAMES**    **NO FRAMES**     **All Classes** |  |
| SUMMARY: NESTED \| FIELD \| CONSTR \| METHOD | DETAIL: FIELD \| CONSTR \| METHOD |  |


---
